# Supplementary material for: Computational Evaluation of N-Based Transannular Interactions in Some Model Fused Medium-Sized Heterocyclic Systems and Implications for Drug Design
Source: Molecules. 2023 Feb 8;28(4):1631. doi: 10.3390/molecules28041631 (PMC9961457; doi:10.3390/molecules28041631)
Supplement: Supplementary file 1 [file molecules-28-01631-s001.zip › molecules-2092984-supplementary.pdf]

Supplementary Materials

Computational Evaluation of N-Based Transannular Interactions in Some Model Fused Medium-Sized Heterocyclic Systems and Implications for Drug Design

Renate Griffith [1,*] and John B. Bremner [2]

[1] School of Natural Sciences (Chemistry), College of Sciences and Engineering, University of Tasmania, Private Bag 75, Hobart, TAS 7001, Australia
[2] School of Chemistry and Molecular Bioscience, Faculty of Science, Medicine and Health, University of Wollongong, Wollongong, NSW 2522, Australia
* Correspondence: renate.griffith@utas.edu.au

Table of Contents:

Calculated structural parameters, relative energies, overlap energies and bond critical densities.

Figure S1. Illustration of structural parameters for N···Y interactions. S1

Table S1. Protopine analogues S2

Table S2. N-substituted protopine compounds S3

Table S3. Imines S4

Table S4. Azo compounds S5

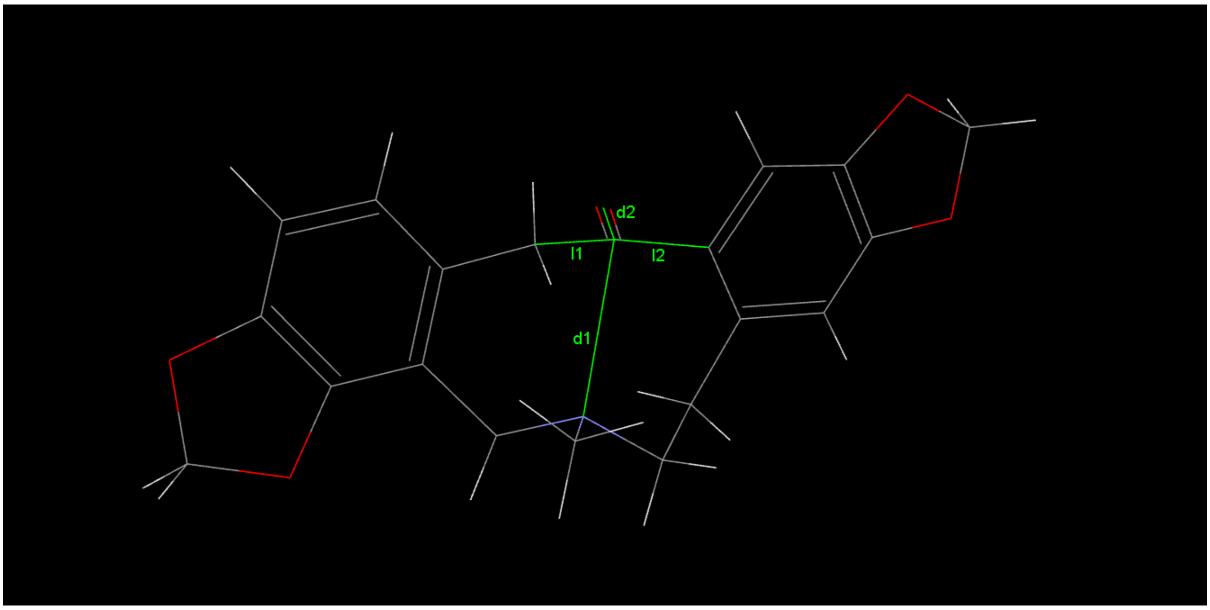

Figure S1. Illustration of structural parameters for N···Y interactions. Illustrated on protopine crystal structure. Other parameters used in tables are: Δ = deviation of Y (carbonyl C for protopine) from plane of its three substituents, measured in Discovery Studio; α = NY angle (NCO for protopine); β_1 = angle between l1 and d2; β_2 = angle between l2 and d2; β_3 = angle between l1 and d1; β_4 = angle between l2 and d1; γ = R-Y-R angle; θ = 120-γ, indicating pyramidalisation of Y.

Table S1. Protopine analogues.

Y*	l_1	l_2	d_1	d_2	Δ	α	β_1	β_2	β_3	β_4	γ	θ	ΔE	Overlap energy N···Y	BCP** density N···Y
Protopine crystal	1.51	1.52	2.55	1.22	0.163	102	120	121	98	85	117	3			
CO; min E	1.53	1.51	2.58	1.21	0.144	102	120	120	63	56	119	1	0	12.29 n-π*	0.023
CO trans pinched			2.46	1.21		97							4	13.84 n-π*	0.028
CS; min E	1.49	1.52	2.67	1.63	0.101	102	121	121	96	83	117	3	0	10.26 n-π*	0.021
CS cis bond	1.52	1.53	1.63	1.78	0.457	110	113	112	104	104	113	7	1.5	No lone pair on N; single bond	0.179
CS trans bond	1.52	1.54	1.61	1.79	0.488	111	111	112	107	105	112	8	2	No lone pair on N; single bond	0.187
BOH cis	1.62	1.62	1.69	1.45	0.413	106	112	110	105	105	117	3	1	No lone pair on N; single bond	0.108
BOH trans	1.62	1.62	1.69	1.45	0.409	105	113	111	106	105	116	4	0	No lone pair on N; single bond	0.109
BOH cis pinched	1.61	1.63	1.80	1.43	0.441	107	115	110	108	104	113	7	3	No lone pair on N; single bond	0.087
O	1.37	1.42	2.84	NA	NA	NA	NA	NA	101	91	118	NA	0	none	0.014
$C{=}CF_2$ min E cis	1.49	1.52	2.82	1.32	0.078	95	118	122	97	83	119	1	0	2.79 n-π*	0.016
S=O min	1.84	1.85	2.83	1.50	0.771	164	105	104	91	75	98	22	0	2.97 n-π*	0.022
$C{=}CH_2$ cis	1.50	1.52	2.81	1.33	0.091	100	120	122	95	81	118	2		3.60 n-π*	0.016
S	1.80	1.84	3.10	NA	NA	NA	NA	NA	88	73	106	NA	0	none	0.015
CH_2	1.52	1.54	3.01	NA	NA	NA	NA	NA	97	82	117	NA		1.40 n-σ*	0.019 (N···H)
CF_2	1.53	1.53	3.06	NA	NA	NA	NA	NA	88	72	120	NA		0.96 n-σ*	0.015 (N···F)

Units: distances in Å; angles in degrees; energies in kcal/mol; densities in atomic units. *: cis, trans refer to relative orientation of N-Me and Y groups. **BCP density = bond critical point density. NA: not applicable.

Table S2. N-substituted protopine compounds.

Y, R*	l_1	l_2	d_1	d_2	Δ	α	β_1	β_2	β_3	β_4	γ	θ	ΔE	Overlap energy N···Y	BCP** density N···Y
CO, CH_3	1.53	1.51	2.58	1.21	0.144	102	120	120	63	56	119	1		12.29 n-π*	0.023
CO, H	1.51	1.53	2.67	1.21	0.136	104	119	120	94	82	119	1	0	8.76 n-π*	0.019
CO, H wide	1.51	1.53	4.15	1.21	0.105	124	119	119	90	62	121	-1	3.51	none	none
CO, OH	1.51	1.53	2.71	1.20	0.136	105	120	120	93	82	120	0	1.19	7.00 n-π*	0.018
CO, OH wide	1.50	1.53	4.15	1.21	0.105	123	119	119	90	62	121	-1	0	none	none
BOH, CH_3	1.62	1.62	1.69	1.45	0.409	105	113	111	106	105	116	4		No lone pair on N; single bond	0.109
BOH, H	1.60	1.63	1.73	1.44	0.419	104	112	114	108	105	113	7		No lone pair on N; single bond	0.098
BOH, OH	1.60	1.63	1.88	1.40	0.388	106	112	115	106	101	114	6		No lone pair on N; single bond	0.073
CS, CH_3	1.49	1.52	2.67	1.63	0.101	102	121	121	96	83	117	3	0	10.26 n-π*	0.021
CS, H	1.49	1.52	2.71	1.63	0.091	103	121	121	96	81	117	3	0.75	7.39 n-π*	0.019
CS, OH	1.50	1.52	2.70	1.62	0.083	101	121	121	96	81	117	3	1.44	6.34 n-π*	0.019
CS, CH_3 bond	1.52	1.54	1.61	1.79	0.488	111	111	112	107	105	112	8		No lone pair on N; single bond	0.187
CS, H bond	1.52	1.54	1.58	1.78	0.464	110	113	114	106	103	110	10	0	No lone pair on N; single bond	0.195
CS, OH bond	1.52	1.54	1.59	1.79	0.476	109	112	113	107	104	111	9	0	No lone pair on N; single bond	0.194
SO, CH_3	1.84	1.85	2.83	1.50	0.771	164	105	104	91	75	98	22		2.97 n-π*	0.022
SO, H	1.84	1.85	2.84	1.50	0.768	165	105	104	90	73	99	21		3.14 n-π*	0.021
SO, OH	1.85	1.86	2.81	1.50	0.777	165	105	104	90	74	98	22		2.82 n-π*	0.022
S, CH_3	1.80	1.84	3.10	NA	NA	NA	NA	NA	88	73	106	NA		none	0.015
S, H	1.79	1.83	3.13	NA	NA	NA	NA	NA	87	71	107	NA			
S, OH	1.79	1.84	3.13	NA	NA	NA	NA	NA	88	74	107	NA			
O, CH_3	1.37	1.42	2.84	NA	NA	NA	NA	NA	101	91	118	NA		none	0.014
O, H	1.37	1.42	2.84	NA	NA	NA	NA	NA	99	88	118	NA			
O, OH	1.37	1.42	2.79	NA	NA	NA	NA	NA	103	96	118	NA			
CH_2, CH_3	1.52	1.54	3.01	NA	NA	NA	NA	NA	97	82	117	NA		1.40 n-σ*	0.019 (N···H)
CH_2, H	1.52	1.54	3.01	NA	NA	NA	NA	NA	96	81	117	NA		1.73 n-σ*	0.018 (N···H)
CH_2, OH	1.52	1.55	2.98	NA	NA	NA	NA	NA	96	82	117	NA		1.35 n-σ*	0.019 (N···H)

Units: distances in Å; angles in degrees; energies in kcal/mol; densities in atomic units. *: R = substituent on N. **BCP density = bond critical point density. NA: not applicable.

Table S3. Imines.

**Y***	**l_1**	**l_2**	**d_1**	**d_2**	**Δ**	**α**	**β_1**	**β_2**	**β_3**	**β_4**	**γ**	**θ**	**ΔE**	**Overlap energy N···Y**	**BCP** density N···Y**
CO trans min	1.53	1.50	2.70	1.21	0.097	110	118	121	82	81	121	-1	0	2.93 n-π*	
CO trans	1.52	1.52	2.55	1.21	0.133	109	119	120	92	79	120	0	4.46	10.63 n-π*	0.023
CO trans2	1.51	1.53	2.93	1.21	0.108	110	118	120	97	70	121	-1	7.40	none	
CO cis	1.50	1.54	4.07	1.21	0.096	133	119	118	90	53	122	-2	3.83	none	
CO cis pinched	1.50	1.53	3.25	1.20	0.131	82	122	121	95	93	117	3	4.71	none	
SO cis	1.80	1.89	3.92	1.49	0.71	106	112	106	77	78	98	22	1.95	none	
SO cis wide	1.82	1.85	4.17	1.50	0.721	158	106	105	86	54	101	19	2.89	none	
SO trans2	1.86	1.86	2.59	1.50	0.791	169	103	105	71	86	97	23	0	4.72 n-π*	0.031
CS trans	1.52	1.50	2.59	1.63	0.090	107	120	121	78	94	118	2	2.20	8.73 n-π*	0.022
CS cis	1.49	1.52	3.22	1.62	0.032	88	123	121	93	91	115	5	0	none	none
BOH trans	1.61	1.63	1.65	1.44	0.589	106	111	115	106	104	113	7	0	No lone pair on N; single bond	0.112
BOH cis	1.57	1.60	3.18	1.36	0.083	84	119	122	94	94	119	1	20.65	none	none
CCH_2 trans	1.50	1.52	2.74	1.33	0.078	105	120	121	93	76	120	0		3.11 n-π*	0.017
CCF_2 trans	1.50	1.52	2.73	1.32	0.066	101	118	121	95	79	121	-1		2.66 n-π*	0.018
O trans	1.37	1.42	2.68	NA	NA	NA	NA	NA	98	86	119	NA		none	0.0173
S trans	1.79	1.83	3.04	NA	NA	NA	NA	NA	83	67	110	NA		none	0.0153
CH_2	1.52	1.54	2.84	NA	NA	NA	NA	NA	93	76	118	NA		0.95;1.03 n-σ* (2xCH)	0.019 (N···H)
CF_2	1.54	1.54	2.89	NA	NA	NA	NA	NA	85	69	122	NA		1.55 n-σ*	0.014 (N···F) 0.019 (N···C5)

Units: distances in Å; angles in degrees; energies in kcal/mol; densities in atomic units. *: cis, trans refer to the orientation of groups around the C=N bond. **BCP density = bond critical point density. NA: not applicable.

Table S4. Azo compounds.

Y*	l_1	l_2	d_1	d_2	Δ	α	$β_1$	$β_2$	$β_3$	$β_4$	γ	θ	ΔE	Overlap energy N···Y	BCP** density N···Y
CO trans	1.52	1.53	2.63	1.21	0.121	111	119	119	92	76	121	-1	2.70	n-π* 6.60	0.019 (N33)
CO trans2	1.51	1.53	2.86 (N39)	1.21	0.100	93	118	119	109	74	122	-2	6.21	none	0.016 (N33)
CO trans min	1.50	1.53	2.68	1.21	0.100	110	121	118	81	81	121	-1	0	n-π* 2.94	0.016 (N33)
CO cis	1.51	1.53	4.19 (N39)	1.21	0.100	147	119	119	94	29	122	-2	8.47	none	none
CS trans	1.50	1.52	2.68 (N33)	1.63	0.075	108	121	120	94	75	118	2	0	n-π* 4.83	0.018 (N33)
CS cis2	1.49	1.52	3.05 (N38)	1.62	0.025	99	123	122	105	67	115	5	1.76	none	none
BOH trans	1.61	1.61	1.67	1.44	0.396	105	115	111	106	103	115	5	0	No LP on N32 (single bond)	0.108 (N32)
BOH cis2	1.57	1.59	2.91	1.35	0.081	91	118	122	108	70	119	1	19.77	none	none
SO trans	1.86	1.86	2.76	1.50	0.765	167	105	104	87	68	99	21	0	n-π* 3.20	0.023
SO cis2	1.81	1.88	4.19 (N37)	1.50	0.744	137	105	104	79	35	101	19	1.19		

Units: distances in Å; angles in degrees; energies in kcal/mol; densities in atomic units. *: cis, trans refer to the orientation of groups around the N=N bond. **BCP density = bond critical point density. NA: not applicable.
